# Widespread hybridization in the introduced hog deer population of Victoria, Australia, and its implications for conservation

**DOI:** 10.1002/ece3.5603

**Published:** 2019-09-04

**Authors:** Erin Hill, Adrian Linacre, Simon Toop, Nicholas Murphy, Jan Strugnell

**Affiliations:** ^1^ Department of Ecology, Environment and Evolution School of Life Sciences La Trobe University Melbourne Vic. Australia; ^2^ College of Science and Engineering Flinders University Adelaide SA Australia; ^3^ Game Management Authority Melbourne Vic. Australia; ^4^ Research Centre for Future Landscapes School of Life Sciences La Trobe University Melbourne Vic. Australia; ^5^ Centre for Sustainable Tropical Fisheries and Aquaculture James Cook University Townsville Qld Australia

**Keywords:** *Axis axis*, *Axis porcinus*, hybridization, introduced species, translocations

## Abstract

In Australia, many species have been introduced that have since undergone drastic declines in their native range. One species of note is the hog deer (*Axis porcinus*) which was introduced in the 1860s to Victoria, Australia, and has since become endangered in its native range throughout South‐East Asia. There is increased interest in using non‐native populations as a source for genetic rescue; however, considerations need to be made of the genetic suitability of the non‐native population. Three mitochondrial markers and two nuclear markers were sequenced to assess the genetic variation of the Victorian population of hog deer, which identified that the Victorian population has hybrid origins with the closely related chital (*Axis axis*), a species that is no longer present in the wild in Victoria. In addition, the mitochondrial D‐loop region within the Victorian hog deer is monomorphic, demonstrating that mitochondrial genetic diversity is very low within this population. This study is the first to report of long‐term persistence of hog deer and chital hybrids in a wild setting, and the continual survival of this population suggests that hybrids of these two species are fertile. Despite the newly discovered hybrid status in Victorian hog deer, this population may still be beneficial for future translocations within the native range. However, more in‐depth analysis of genetic diversity within the Victorian hog deer population and investigation of hybridization rates within the native range are necessary before translocations are attempted.

## Graphical abstract

The hog deer (*Axis porcinus*) was introduced in the 1860s to Victoria, Australia, and has since become endangered in its native range throughout South‐East Asia. There is some belief that this population could be a source for genetic rescue within the native range, so three mitochondrial and two nuclear markers were sequenced to identify which species of hog deer was introduced and to assess the genetic variation of the Victorian population. It was discovered that this population has hybrid origins with the closely related chital (*Axis axis*) and comprises low genetic diversity at the D‐loop region. Although this population is comprised of hybrids, it may still be beneficial for the genetic rescue of native hog deer; however, more research into the hybridization rates in the native range is now needed.

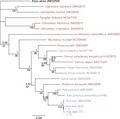

## 1 INTRODUCTION

The introduction and successful establishment of non‐native populations of threatened species is becoming increasingly common. Today, this occurs frequently as an impact of illegal wildlife trade, but past introductions of common species that have subsequently undergone declines in their native range are also increasingly being recognized (Gibson & Yong, 2017). These non‐native populations are steadily being acknowledged for their potential to be used for conservation programs in the form of translocations back into native ranges (Garzón‐Machado, del‐Arco‐Aguilar, & Pérez‐de‐Paz, 2012; Marchetti & Engstrom, 2016). These translocations can lead to the genetic rescue of native populations, which can alleviate negative genetic impacts associated with small, isolated populations, such as low genetic diversity, increased genetic drift, and inbreeding depression (Weeks et al., 2011). However, using non‐native populations of threatened species for genetic rescue is likely to come with challenges; founder effects are commonly encountered in introduced populations, presenting a loss of genetic diversity from the native population, and an increase in genetic drift (Frankham et al., 1999). Nevertheless, recent investigations of non‐native populations of a number of threatened species show only minor differences in genetic variation between introduced and native populations, highlighting that these perceived negative genetic impacts associated with introduced populations are not always present, especially if insufficient time has passed following introductions for genetic drift and divergence to occur, or if multiple introductions of the same species have occurred (Collins, Freeman, & Snow, 2008; Vörös, Mitchell, Waldman, Goldstien, & Gemmell, 2008). These factors can vary across introduced populations/species owing to life‐history traits and time since introduction, and therefore, the need to evaluate the suitability of threatened, non‐native populations for their potential as candidates for translocations needs to be considered on a case‐by‐case basis.

In Australia, several introduced species, particularly from the Order Artiodactyla, have since become threatened with extinction in their native range; the IUCN lists both sambar (*Rusa unicolor*) and Javan rusa deer (*Rusa timorensis*) as vulnerable, while banteng (*Bos javanicus*) and hog deer (*Axis porcinus*) are listed as endangered in their native range. The use of the Australian banteng population for conservation has been explored in the past (Bradshaw, Isagi, Kaneko, Bowman, & Brook, 2006; Bradshaw et al., 2007); however, little attention has been given to the hog deer population present in Victoria. The hog deer is native to South‐East Asia and is present in Pakistan, India, Nepal, and Bangladesh, as well as small, introduced populations in Sri Lanka and the United States (Timmins et al., 2015). Declines throughout their native range have been associated with overhunting, predominantly for meat, trophies, and velvet antler used in traditional medicine, and conversion of their preferred habitat of tall floodplain grasslands for agriculture and commercial development (Timmins et al., 2015). The largest non‐native population of hog deer exists in Australia, where a stable, continuous population occurs throughout the Gippsland region in Victoria (Scroggie, Forsyth, & Brumley, 2012). Hunting of hog deer is restricted to April every year, and only one male and one female may be harvested per season per hunter, in part because the population is thought to be important for the long‐term conservation of the species. Assessing the potential for the hog deer in Victoria to be used for conservation efforts is therefore not only important for the declining populations within the native range, but also for the management of the deer in Victoria. Negative impacts to native flora and fauna due to deer damage are well known, and hog deer can therefore be managed more effectively to mitigate these impacts if their conservation worth is effectively evaluated (Davis et al., 2016; Davis, Coulson, & Forsyth, 2008; Davis, Forsyth, & Coulson, 2010).

There are a number of considerations to be made when assessing the Victorian hog deer's suitability for translocations as part of conservation programs. Firstly, it is important to identify which subspecies has established in Victoria. The Acclimatisation Society of Victoria brought hog deer from South‐East Asia to Victoria in the 1860s, primarily sent from ports in Sri Lanka and India (Mayze & Moore, 1990). The Sri Lanka population itself is thought to be comprised of introduced hog deer of unknown origin (Timmins et al., 2015). Furthermore, following these introductions to Australia, a new subspecies of hog deer was described in South‐East Asia, the Indochinese hog deer (*Axis porcinus annamiticus*: Heude 1888). Today, this subspecies is considered critically endangered and occurs in isolated populations in Cambodia and Thailand; however, in the past its distribution was more widespread (Brook, Nask, & Channa, 2015; Maxwell, Nareth, Kong, Timmins, & Duckworth, 2006). Hunters in Victoria have also previously reported two distinct “forms” of hog deer found throughout Gippsland, with one form described as being smaller with a stockier build (Bentley, 1978).

The second consideration when evaluating the Victorian hog deer population for translocations is the possibility that either past or contemporary hybridization has occurred. Hybridization is prolific within the family Cervidae, and hybrid zones have been recorded in the genera *Cervus* (Lowe & Gardiner, 1975; McDevitt et al., 2009; Moore & Littlejohn, 1989; Senn & Pemberton, 2009), *Odocoileus* (Ballinger, Blankenship, Bickham, & Carr, 1992; Carr, Ballinger, Derr, Blankenship, & Bickham, 1986; Cathey, Bickham, & Patton, 1998), and most recently in *Rusa* (Martins, Schmidt, Lenz, Wilting, & Fickel, 2018). While hybridization between species within the *Axis* genus has not been reported in the wild, there have been cases of chital (*Axis axis*) and hog deer hybrid offspring being born in captivity, with animals from the two species needing to be separated due to their proclivity to interbreed (Gray, 1972; Mayze & Moore, 1990; McMaster, 1871). Chital have been released in Victoria in the past, including in areas where hog deer are now known to occur; however, these chital populations have since become locally extirpated (Forsyth, Stamation, & Woodford, 2016). Many species of deer were housed together in Royal Park (now the Royal Melbourne Zoological Gardens) prior to release (“A Few Hours in the Zoological and Acclimatisation Society's Grounds” 1873; “The Naturalist”, 1873), so it is possible that hog deer and chital were housed in captivity together prior to their liberation. Additionally, previous research has shown that while native hog deer and chital mitochondrial genomes share a 94.65% identity, the mitochondrial genomes of Victorian hog deer and chital show a greater degree of similarity which may indicate hybridization; however, this was detected in only four samples from a managed island population in Victoria, and so may not be representative of the entire population (Hassanin et al., 2012; Hill, Linacre, Toop, Murphy, & Strugnell, 2017). There are also unconfirmed reports that another species from the *Axis* genus, the Bawean hog deer (*Axis kuhlii*), was introduced to Victoria and possibly released; however, there is some debate that the species introduced was actually the Javan rusa (Bentley, 1978; Mayze & Moore, 1990). Today, the hog deer range in Victoria overlaps with both sambar and fallow deer (*Dama dama*; Forsyth, Stamation, & Woodford, 2015; Forsyth et al., 2016). While hybridization across two different genera of deer is likely to be rare, hybridization between fallow and hog deer has been recorded in captivity in the past, although it is unknown how long the offspring survived, or if they were fertile (Gray, 1972).

The final consideration necessary to evaluate the suitability of hog deer for conservation efforts is to estimate the genetic diversity present within the Victorian population. When choosing individuals to use for translocations, it has been suggested that capturing >95% of the genetic variation present in the source population is necessary in order to offset the negative genetic impacts present in the receiving population (Weeks et al., 2011). The founding population of hog deer in Victoria was only comprised of 15 individuals (nine females, two males, and four of unknown sex); however, the population has thrived since being released, and a continuous breeding population is present across a 2,336 km^2^ range (Forsyth et al., 2016; Mayze & Moore, 1990). In order to capture as much genetic diversity as possible, it is crucial to sample individuals throughout the entire range in Victoria to determine how much variation is present in the population, and if particular sites contain greater diversity and are therefore more suitable for translocation.

Genetic analyses of mitochondrial and nuclear DNA are able to address the suitability of the Victorian population of hog deer as a potential source population for conservation efforts of the species. Mitochondrial markers, and the nuclear markers alpha‐lactalbumin (αLalb) and protein kinase C iota I (PRKCI), have been used in the past to investigate the phylogeny and genetic diversity of various deer species (Ludt, Schroeder, Rottmann, & Kuehn, 2004; Vernesi et al., 2002) and have been shown to be effective at delineating species (Hassanin & Douzery, 2003; Ropiquet & Hassanin, 2005). These markers will be utilized in this study to first establish which species or subspecies of hog deer was introduced to Australia, and then to assess the genetic diversity and any occurrence of hybridization within this population in order to ascertain the value of the Victorian population as a source for genetic rescue within the native range.

## 2 METHODS

### 2.1 Sampling and DNA extraction

**Figure 1:**
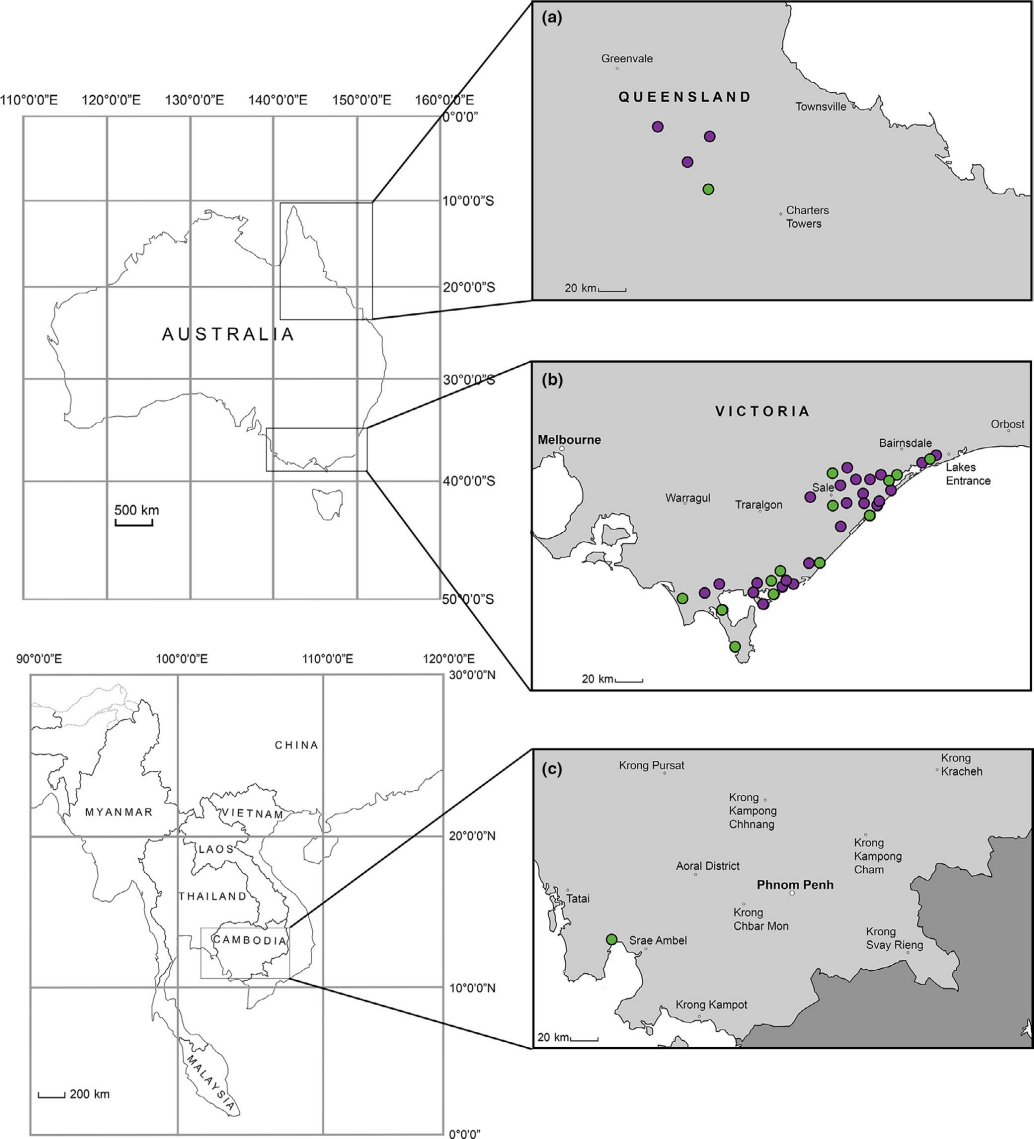
Maps showing the collection sites for samples used for sequencing analysis in this study: (a) chital (*Axis axis*) sampling locations in Queensland, Australia; (b) hog deer (*Axis porcinus*) sampling locations in Victoria, Australia; and (c) Indochinese hog deer (*Axis porcinus annamiticus*) sampling location in Koh Kong Province, Cambodia. Green circles indicate sites where sequences for all five genes were obtained, and purple circles indicate sites where only the D‐loop was sequenced

A total of 78 liver and tongue samples were collected from wild, free‐ranging hog deer (presumed *Axis porcinus*) during the hunting seasons from 2015 to 2017, in Victoria (VIC), Australia (Figure 1). Museum Victoria provided a further two voucher samples of Australian hog deer, collected during a deer cull at Wilsons Promontory National Park in 2015 (Museum No. Z52238 and Z52239). Additionally, samples were collected from two deer species also belonging to the genus *Axis*. These included 35 tissue samples from the wild, free‐ranging chital (*Axis axis*), collected in Queensland (QLD), and two skin samples from the Indochinese hog deer (*Axis porcinus annamiticus*), collected from Cambodia (KHM) in the Koh Kong Province (Figure 1). Extractions of tissue samples were carried out using a DNeasy Blood and Tissue Kit (Qiagen), following the manufacturer's instructions, and DNA was quantified using a Qubit 2.0 Fluorometer (Invitrogen).

### 2.2 PCR amplification

Three mitochondrial markers and two nuclear markers were used in this study. Universal markers for the mitochondrial genes, cytochrome b (Cyt *b*) and cytochrome oxidase subunit I (COI), were utilized, as well as custom‐made D‐loop primers (Branicki, Kupiec, & Pawlowski, 2003; Folmer, Black, Hoeh, Lutz, & Vrijenhoek, 1994; Kocher et al., 1989; Table 1). Nuclear regions of intron 2 of the α‐lactalbumin gene (αLalb) and the intron of protein kinase C iota (PRKCI) were also sequenced (Hassanin & Douzery, 2003; Ropiquet & Hassanin, 2005; Table 1). Twenty‐two samples of VIC hog deer and five samples of QLD chital were sequenced for the above‐described genes. All regions were also sequenced for one *Axis porcinus annamiticus* sample, except the PRKCI gene which failed to amplify. The samples of hog deer selected for sequencing of all five gene regions were chosen to represent the spatial distribution of the hog deer population in Victoria. A further 58 samples of hog deer, 30 samples of chital, and one additional sample of *A. p. annamiticus* were sequenced for the D‐loop region to assess the genetic diversity of the populations. This region was chosen to further investigate genetic diversity as the mitochondrial control region, where the D‐loop is located, is considered to be the most polymorphic section of the mitochondrial genome, and several studies have utilized the D‐loop to assess genetic diversity in several deer species in the past (Hu, Fang, & Wan, 2006; Moritz, Dowling, & Brown, 1987; Pérez‐Espona et al., 2009; Skog et al., 2009).

**Table 1:** Gene regions and primers used to generate sequence data for this study

Gene region	Primer name	Primer sequence (5′–3′)	*n* (Hog deer, Chital, *A. p. annamiticus*)	Fragment length	Reference
COI	LCO1490	GGTCAACAAATCATAAAGATATTGG	22, 5, 1	567 bp	Folmer et al. (1994)
	HCO2198	TAAACTTCAGGGTGACCAAAAAATCA			
Cyt *b*	L14724	CGAAGCTTGATATGAAAAACCATCGTTG	22, 5, 1	387 bp	Kocher et al. (1989)
	H15149	AAACTGCAGCCCCTCAGAATGATATTTGTCCTCA			
D‐loop	FD15378	CCTAAGACTCAAGGAAGAAGCCATA	80, 35, 2	576 bp	This study
	R16130	GATGCAGTTAAGTCCAGCTACAATT			
αLalb	αLalbF	ATCTGTAACATCTCCTGTGA	22, 5, 1	382 bp	Hassanin and Douzery (2003)
	αLalbR	TCAGTAAGRTCATCATCCAG			
PRKCI	U26	TATGCTAAAGTACTGTTGGT	22, 5, –	412 bp	Ropiquet and Hassanin (2005)
	L748	CTGTACCCAGTCAATATCCT			

Sequence PCRs were carried out in 25 μl reactions, consisting of 12.5 μl of MyTaq™ Red Mix (Bioline), 0.5 μl (10 μM) of forward primer, 0.5 μl (10 μM) of reverse primer, 1.5 μl of MgCl_2_, 8 μl of H_2_O, and 2 μl of template DNA, ranging from 3.69 to 317 ng/μl. Cycling conditions were the same for each gene region, with an initial denaturation at 95°C for 15 min, 40 cycles of 94°C for 30 s, 60°C for 30 s, 72°C for 1 min, followed by a final extension of 72°C for 5 min. PCR products were visualized on a 1% agarose gel and sent to Macrogen Inc. (South Korea) for sequencing.

### 2.3 Data analysis

#### 2.3.1 Phylogenetics

Sequences were aligned using the Geneious alignment method in *Geneious 9.0.5* (Kearse et al., 2012). Additional sequences of hog deer and chital were downloaded from GenBank to include in the dataset (Table 2). Further sequences of all deer species that have been introduced to Australia, including those that did not become established, were also included in the dataset where available (Table 2). Sequences of moose (*Alces alces*) were used as an outgroup for phylogenetic analyses. These sequences were trimmed and aligned with the hog deer, chital, and Indochinese hog deer sequences. Mitochondrial genes were concatenated for analyses, while the nuclear genes αLalb and PRKCI were analyzed separately. Alignments were run in *JModelTest 2.1.7* to determine the best‐fit model for each alignment, using the Akaike information criterion (Akaike, 1974; Darriba, Taboada, Doallo, & Posada, 2012). Best‐fit models were GTR + I + G for the concatenated mitochondrial alignment, TrN + G for the αLalb gene, and F81 + G for the PRKCI gene. Bayesian phylogenetic analyses were run in *MrBayes 3.2.6* to calculate posterior probabilities, which were run for 1,000,000 generations, after a burn‐in period of 100,000, and sampling trees every 200 generations (Huelsenbeck & Ronquist, 2001). This analysis was performed twice for every gene, to ensure convergence toward the same likelihood score. Maximum likelihood phylogenetic trees were created using the program *PHYML*, using the same models described previously, with 1,000 bootstrap replicates (Guindon et al., 2010). The program *PAUP 4.0* was used to calculate genetic distances for the concatenated mitochondrial tree, using the model identified in *JModelTest 2.1.7* (Swofford, 1999).

**Table 2:** Sequences obtained from GenBank that were combined with the main dataset

Latin name	Common name	Mitochondrial	aLalb	PRKCI
*Axis axis*	**Chital**	JN632599 a	DQ379348 c	DQ379329 c
*Axis kuhlii*	Bawean hog deer	−	−	−
*Axis porcinus*	**Hog deer**	JN632600 a	DQ379349 c	DQ379367 c
*Capreolus capreolus*	Roe deer	JN632610 a	AY122021 d	DQ365692 c
Cervus canadensis songaricus*	Wapiti/Elk	KJ025072 b	−	−
*Cervus elaphus*	**Red deer**	NC007704	AY122017 d	AY846793 d
*Cervus nippon*	Sika deer	AB211429	DQ379352 c	DQ379332 c
*Dama dama*	**Fallow deer**	JN632629 a	DQ379356 c	DQ379335 c
*Hydropotes inermis*	Chinese water deer	JN632649 a	AY122020 d	DQ379340 c
Moschus moschiferus*	Musk deer	JN632662 a	AY122033 d	DQ365693 c
*Muntiacus muntjak*	Indian muntjac	NC004563	−	−
*Odocoileus hemionus*	Mule deer	JN632670 a	AY122022 d	DQ379345 c
*Odocoileus virginianus*	White‐tailed deer	JN632672 a	DQ379365 c	DQ379346 c
*Rangifer tarandus*	Reindeer	NC007703	AY122019 d	AF165693 e
*Rucervus duvaucelii*	Barasingha	JN632696 a	DQ379351 c	DQ379331 c
*Rucervus eldii*	Eld's deer	JN632697 a	DQ379353 c	−
*Rusa timorensis*	**Javan rusa deer**	JN632699 a	DQ379354 c	DQ379333 c
*Rusa unicolor*	**Sambar deer**	NC008414	DQ379355 c	DQ379334 c

Note

These species were all historically introduced to Australia (present‐day species in Australia in bold), as reported by Moriarty (2004). Sequences could not be found for *C. canadensis* and *Moschus sibiricus*; so, closely related species were used as a substitute (asterisk).

a Hassanin et al. (2012).

b Li, Ba, and Yang (2016).

c Gilbert et al. (2006).

d Hassanin and Douzery (2003).

e Matthee, Burzlaff, Taylor, and Davis (2001).

#### 2.3.2 Population genetics

Sequences of the D‐loop region were trimmed to 576 bp and aligned using the Geneious alignment method in *Geneious 9.0.5* (Kearse et al., 2012). Measures of haplotype diversity and nucleotide diversity were calculated for the VIC hog deer and QLD chital using *DNAsp 5.10.1* (Librado & Rozas, 2009). The neutrality tests Tajima's *D* and Fu's Fs were calculated for each population in the program *Arlequin 3.5* (Excoffier & Lischer, 2010; Fu, 1997; Tajima, 1989). Samples collected in this study were then trimmed to 372 bp to align with native samples of chital, *A. porcinus*, and *A. p. annamiticus* downloaded from GenBank in order to create a haplotype network (Table 3). Samples of hog deer taken from GenBank were assigned to either *A. porcinus* or *A. p. annamiticus* based on the species names reported in GenBank. A median‐joining haplotype network was generated using the program *PopART* (Leigh & Bryant, 2015). Populations of native chital, native *A. porcinus*, and native *A. p. annamiticus* (including two *A. p. annamiticus* samples collected in this study) were also analyzed for the genetic diversity and neutrality indices described above.

**Table 3:** D‐loop sequences of chital (*Axis axis*), *A. porcinus*, and *A. p. annamiticus* taken from GenBank that were combined with the main dataset

Species	Accession no.	Location	Reference
*Axis axis*	JN596132–JN596143	India	Direct submission
	JN596145–JN596148		
	JN596150–JN596151		
	JN596153–JN596156		
	JN632599	Captive	Hassanin et al. (2012)
*Axis porcinus*	EF491198–EF491199	Thailand	Direct submission
	EF491201–EF491202		
	EU870592	India	Direct submission
	JN632600	Captive	Hassanin et al. (2012)
	MH392156–MH392168	India	Gupta et al. (2018)
*Axis porcinus annamiticus*	KM881614–KM881625	Thailand	Direct submission

## 3 RESULTS

### 3.1 Phylogenetics

**Figure 2:**
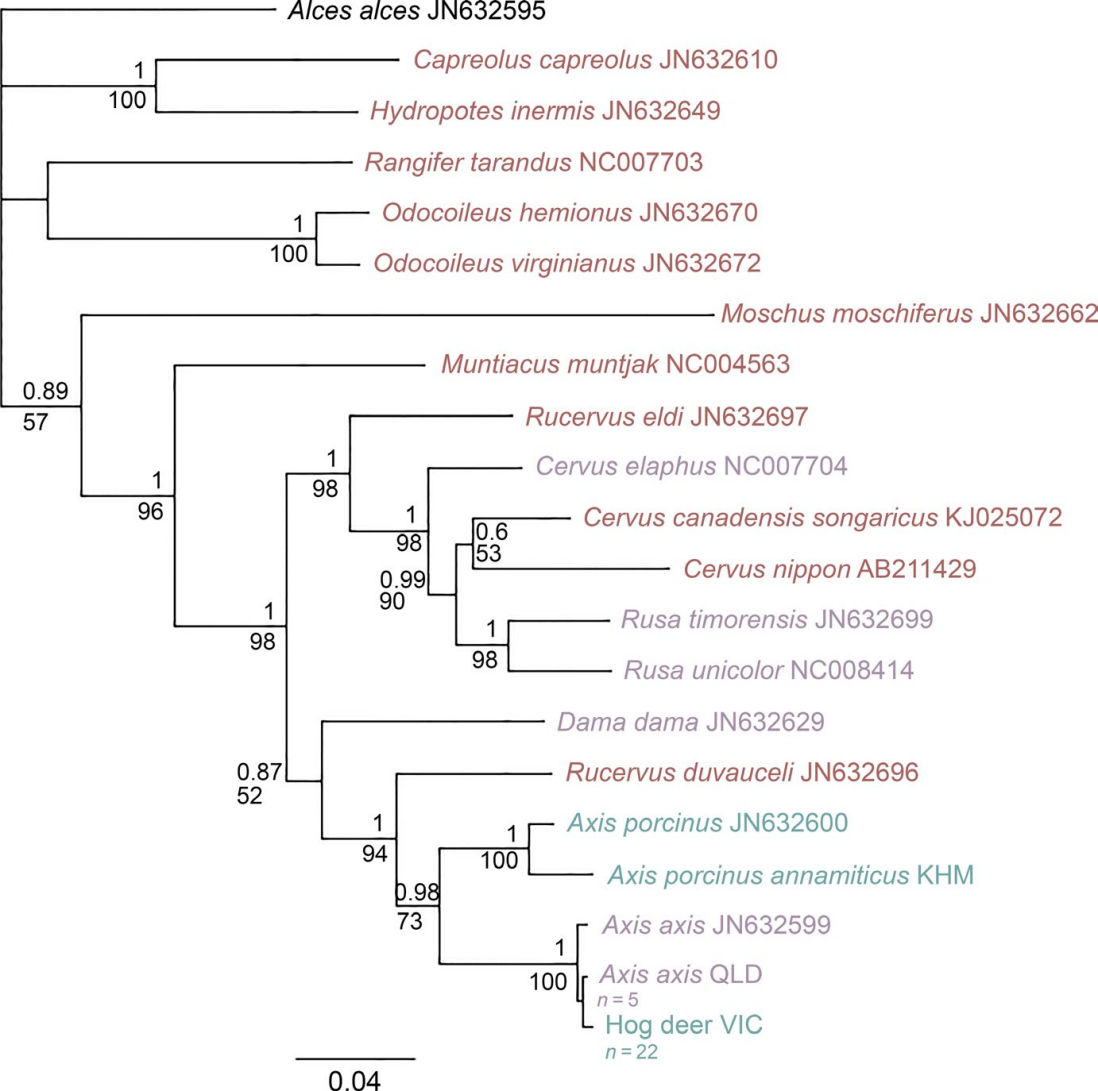
Bayesian phylogenetic tree based on 1,530 bp concatenated mtDNA regions of COI, Cyt *b*, and the D‐loop. Bayesian posterior probability values are reported above each node; maximum likelihood bootstrap values are reported below the nodes. GenBank accession numbers are written next to species names. Blue text indicates hog deer species; purple text indicates deer species currently established in Australia; and red text indicates deer species historically introduced to Australia. Sample sizes are specified where multiple samples shared the same haplotype. *Alces alces* was used as an outgroup

All unique sequences generated in this study for each species have been deposited in GenBank (accession no. MN226858–MN226880). A 567‐base‐pair (bp) region of the COI gene, a 387‐bp region of the Cyt *b* gene, and a 576‐bp fragment of the D‐loop region were successfully amplified in each of the 22 Victorian hog deer, five Queensland chital, and one Cambodian Indochinese hog deer samples, to create a concatenated 1,530‐bp mitochondrial fragment. Topologies from the Bayesian analysis of the mitochondrial data show that both VIC hog deer and QLD chital fall within a single clade, along with one sample of *Axis axis* taken from GenBank (accession no. JN632599; Figure 2). This clade was highly supported, with posterior probability (PP) and bootstrap (BS) values being 1 and 100, respectively. All samples of QLD chital were shown to share the same haplotype at the three mitochondrial genes, while samples of VIC hog deer also comprised a single haplotype for the three mitochondrial genes. The sample of Indochinese hog deer was shown to form a separate clade with the sample of *Axis porcinus* available in GenBank (JN632600), again with highly supported PP and BS values.

**Figure 3:**
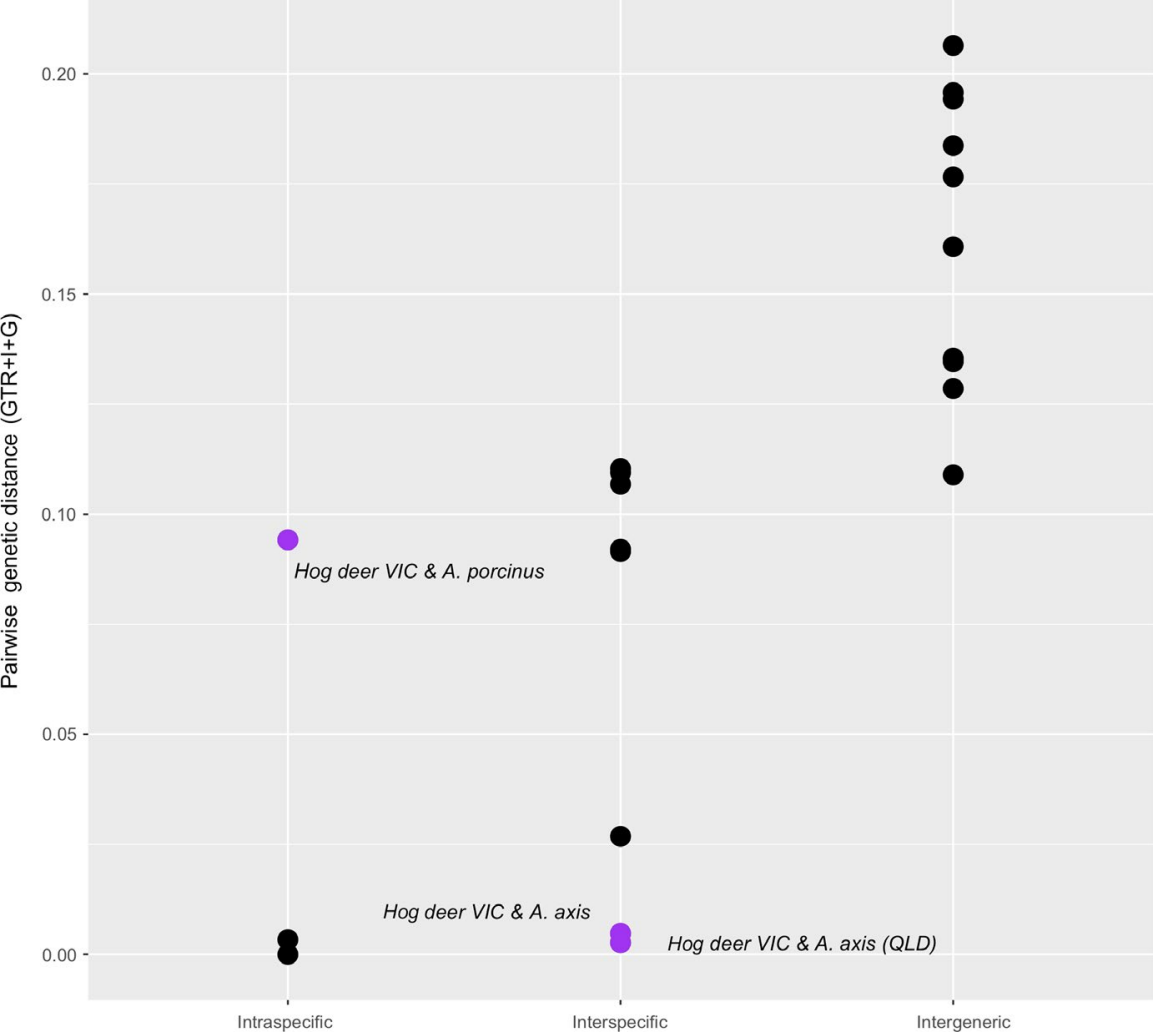
mtDNA pairwise distances (GTR + I + G) within hog deer and chital species (intraspecific), between separate species in the *Axis* genus (interspecific), and between species belonging to different genera (intergeneric), which includes samples of *Dama dama* and *Rucervus duvauceli*. Data points highlighted in purple represent pairwise comparisons between Victorian hog deer and *Axis porcinus* and *Axis axis* samples

Genetic distances calculated between the VIC hog deer and the QLD and GenBank chital reveal a distance of 0.003–0.005 (Table 4), considerably lower than observed for interspecific genetic distances between other deer species in the *Axis* genus (Figure 3). Interspecific genetic distances between other species in this genus ranged from 0.03 to 0.11, with the lowest value representing the difference between *Axis porcinus* and the subspecies *Axis porcinus annamiticus*. Conversely, genetic distances between the VIC hog deer and the other hog deer species were 0.09 and 0.11 (*Axis porcinus* and *Axis porcinus annamiticus*, respectively), notably higher than expected when compared to intraspecific genetic distance within the *Axis* genus (range from 0 to 0.003).

**Table 4:** Pairwise genetic distances for all species of hog deer and chital, based on the concatenated mitochondrial sequences, using the evolutionary model GTR + I+G

	Hog deer VIC	*A. porcinus*	*A. p. annamiticus*	*A. axis* QLD	*A. axis*
Hog Deer VIC					
*A. porcinus*	0.09				
*A. p. annamiticus*	0.11	0.03			
*A. axis* QLD	0.003	0.06	0.11		
*A. axis*	0.005	0.09	0.11	0.003	

**Figure 4:**
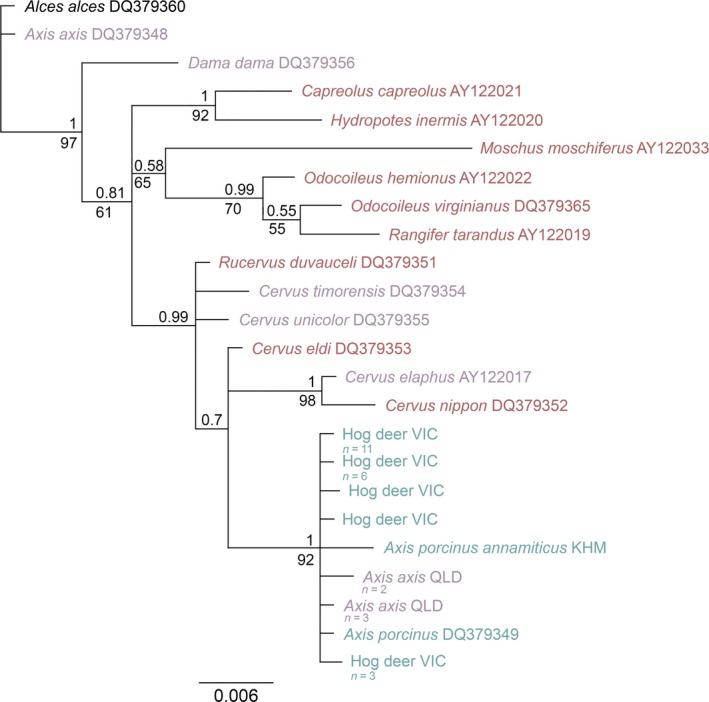
Bayesian phylogenetic tree based on 382 bp of the αLalb gene. Bayesian posterior probability values are reported above each node; maximum likelihood bootstrap values are reported below the nodes. GenBank accession numbers are written next to species names. Blue text indicates hog deer species; purple text indicates deer species currently established in Australia; and red text indicates deer species historically introduced to Australia. Sample sizes are specified where multiple samples shared the same haplotype. *Alces alces* was used as an outgroup

A 382 bp of the αLalb region and a 412‐bp region of the PRKCI gene were successfully amplified from each sample of this study. Phylogenetic analyses of the αLalb gene revealed that a polytomy was formed with the Victorian hog deer, the Queensland chital samples, *Axis porcinus annamiticus*, and an *Axis porcinus* sample taken from GenBank (Figure 4). This clade was highly supported by both PP and BS values (1 and 92, respectively). A total of five haplotypes were identified in the Victorian hog deer population, and 11 of these samples shared the same haplotype as *Axis porcinus* DQ379349. The chital samples from Queensland appear to be more distinct from the hog deer samples while still belonging to the same group; however, these samples did not cluster with the GenBank sample of *Axis axis*. The *Axis axis* DQ379348 sample is shown to form a clade with *Axis porcinus* DQ379349 in Gilbert, Ropiquet, and Hassanin (2006); however, in the present study *Axis axis* DQ379348 formed a clade with the outgroup (*Alces alces*) and was shown to be identical, suggesting that this sample has been mislabelled in GenBank and is likely to be *Alces alces*.

**Figure 5:**
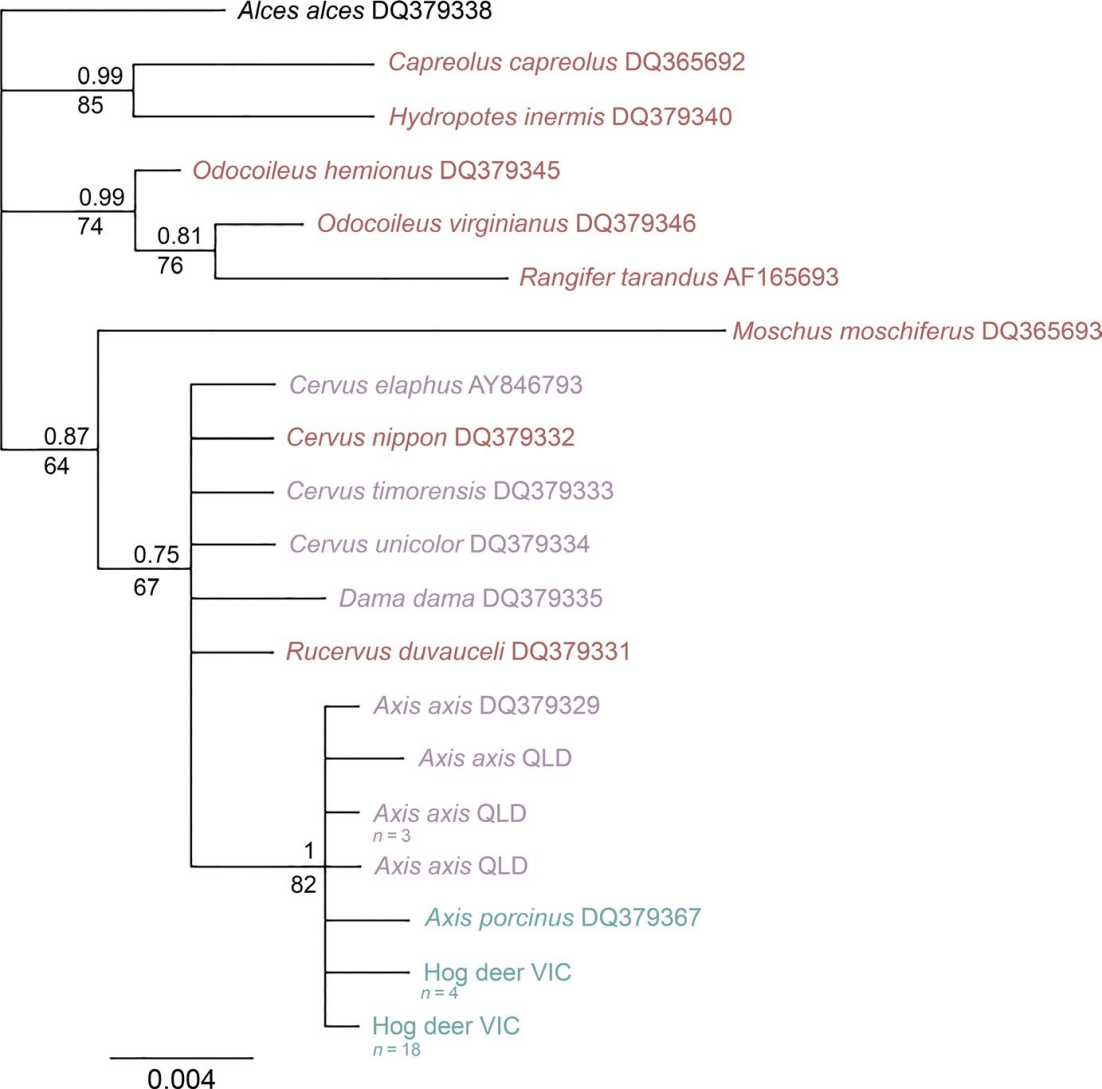
Maximum likelihood phylogenetic tree based on 412 bp of the PRKCI gene. Bayesian posterior probability values are reported above each node; maximum likelihood bootstrap values are reported below the nodes. GenBank accession numbers are written next to species names. Blue text indicates hog deer species; purple text indicates deer species currently established in Australia; and red text indicates deer species historically introduced to Australia. Sample sizes are specified where multiple samples shared the same haplotype. *Alces alces* was used as an outgroup

Topologies of the PRKCI Bayesian analyses again reveal that the Victorian hog deer samples are within the same group as the *Axis axis* samples, forming a polytomy with *Axis axis* DQ379329 and *Axis porcinus* DQ379367 (Figure 5). All samples within this polytomy are highly supported, with PP and BS values of 1 and 82, respectively. Two haplotypes are present in the Victorian hog deer, with the most common of the two being shared with one Queensland chital sample and a GenBank *Axis axis* sample. The second VIC hog deer haplotype, the remaining Queensland chital samples, and the GenBank *Axis porcinus* sample all form separate, distinct branches within this group.

#### 3.1.1 Population genetics

A 576‐bp fragment of the D‐loop was successfully sequenced in 35 chital from QLD, 80 hog deer from VIC, and two samples of *A. p. annamiticus* from Cambodia. A single haplotype was identified in the hog deer population, indicating that the population is monomorphic for this gene. The QLD chital population comprises two distinct haplotypes, with a haplotype diversity of 0.461 (±0.07 *SD*) and a nucleotide diversity of 0.0023 (±0.00035 *SD*; Table 5). In comparison, much greater diversity was detected in the native chital and hog deer populations sourced from GenBank. The number of observed haplotypes ranged from 12 in the native *A. p. annamiticus* samples to 17 in the *A. porcinus* group. The number of polymorphic sites was also much higher, with 21 observed in the native chital, and 26 observed in both the *A. porcinus* and *A. p. annamiticus* samples. Haplotype diversity ranged from 0.913 (±0.04 *SD*) in the native chital to 0.988 (±0.02 *SD*) in the native *A. porcinus*, while nucleotide diversity ranged from 0.017 (±0.0015 *SD*) to 0.023 (±0.0019 *SD*) in the native populations (chital and *A. porcinus*, respectively). Neutrality tests were not significant in any of the populations.

**Table 5:** Genetic diversity measurements based on a fragment of the D‐loop for the hog deer population in Victoria, the chital population in Queensland, and samples from the native range of chital, *Axis porcinus*, and *Axis porcinus annamiticus*

	Native *A. porcinus*	Native *A. p. annamiticus*	Hog deer VIC	*A. axis* QLD	Native *A. axis*
*n*	19	14	80	35	23
*N*_h_	17	12	1	2	14
*S*	26	26	0	4	21
*h* (±*SD*)	0.988 (0.02)	0.978 (0.04)	0	0.461 (0.07)	0.913 (0.04)
*π* (±*SD*)	0.023 (0.0019)	0.019 (0.0062)	0	0.0023 (0.0004)	0.017 (0.0015)
Tajima's *D*	−0.290	−0.560	–	0.880	−0.466
*P*_TD_	0.43	0.31	–	0.82	0.39
Fu's Fs	−1.267	2.151	–	2.593	0.830
*P*_FS_	0.26	0.82	–	0.89	0.66

Note

*n*, sample size; *N*_h_, number of haplotypes; *S*, number of polymorphic sites; *h*, haplotype diversity; *π*, nucleotide diversity; *SD*, standard deviation; *P*_TD_, probability of Tajima's *D*; *P*_FS_, probability of Fu's Fs.

**Figure 6:**
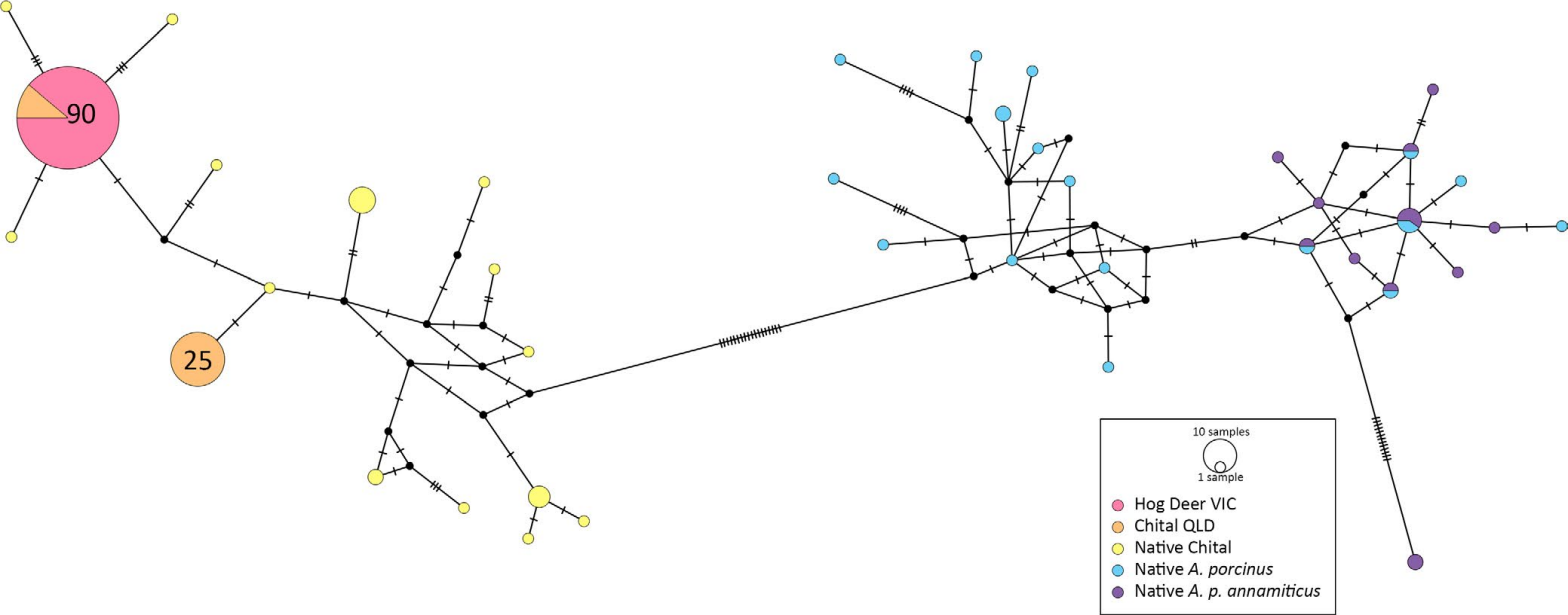
Median‐joining haplotype network based on the mitochondrial D‐loop, comprising Victorian hog deer, Queensland chital, and native *Axis axis*, *Axis porcinus*, *and Axis porcinus annamiticus* samples. Circle size is indicative of the frequency of each haplotype. Numbers indicate sample sizes for common haplotypes. Hatch marks represent the number of bp changes between haplotypes. Black circles represent median vectors

The median‐joining haplotype network created with the combined chital and hog deer data generated in this study and taken from GenBank shows four distinct groups within the data (Figure 6). Only two haplotypes are present in the Australian populations, both of which are found in the broader chital group. One of these haplotypes is shared between the VIC hog deer and QLD chital, with all hog deer individuals and 10 chital samples containing this haplotype. The haplotypes detected in Australian samples of hog deer and chital were not observed in the native chital samples; however, for both Australian haplotypes, only one bp difference is observed between these samples and the nearest related native chital sample. The native hog deer samples separated into three groups: an *A. porcinus* group, an *A. p. annamiticus* group, and a mixed group comprising samples from both subspecies. Only two samples were present in the *A. p. annamiticus* group, which were both sequenced in the present study. All other samples belonging to *A. p. annamiticus* as reported in GenBank were clustered into the mixed group with samples identified as *A. porcinus*, with four haplotypes being shared between the two subspecies.

## 4 DISCUSSION

The mitochondrial and nuclear data presented here portray an interesting insight into the recent genetic history of hog deer in Victoria, Australia. Due to the discovery of chital haplotypes in the mitochondrial DNA of hog deer in Victoria, it is not possible to identify which species/subspecies of hog deer was initially introduced using traditional barcoding methods. Results from the nuclear gene region αLalb indicate that these haplotypes are more closely related to *Axis porcinus* rather than *Axis porcinus annamiticus*, which may suggest that the species introduced to Australia was the Indian hog deer *A. porcinus*. However, given the low levels of resolution at this nuclear marker, additional analysis using more variable nuclear STRs or SNPs is needed to firmly conclude which hog deer species was released in Victoria.

The mitochondrial data showed that hog deer in Victoria possess haplotypes that are most closely allied with the chital, a species that historically was released and established a population in Victoria but has since become locally extirpated (Forsyth et al., 2016). This may be a result of incomplete lineage sorting; however, it is more likely that hybridization has occurred between these two species. The time of divergence between chital and *Axis porcinus* occurred during the Pliocene, approximately 2.6 Mya, and a number of mitochondrial haplotypes exclusive to each species have been detected, as seen in the haplotype network presented in this study (Gilbert et al., 2006; Gupta et al., 2018; Hassanin et al., 2012). The presence of these species‐specific haplotypes suggests that incomplete lineage sorting is unlikely to be a factor influencing the mitochondrial results reported here. Hog deer and chital are also morphologically distinct; mature hog deer have a stocky build, reach 60–70 cm in shoulder height, and have a reddish‐brown coat color with a dark dorsal stripe across the back of the neck and spine, with light‐colored spots along either side of the dorsal stripe. Chital are larger, finer in build with a shoulder height of 70–90 cm, and a reddish‐brown coat color covered in distinct white spots. These differences, along with the absence of a wild chital population in Victoria, highlight that the results reported here are unlikely to be due to misidentification based on morphological appearance. Additionally, hybrids between captive hog deer and chital have been recorded previously. These have typically arisen from the mating of a male hog deer with a female chital, as seen in the present study with the presence of chital haplotypes in the maternally inherited mitochondrial genome. Similar unidirectional hybridization has been reported in red deer and sika deer hybrids, with genetic contributions from female red deer and male sika deer being reported a majority of the time when analyzed throughout Ireland and the UK (Smith, Carden, Coad, Birkitt, & Pemberton, 2014; Smith et al., 2018). In both cases, hybridization occurs between the female of the larger deer species (chital and red deer) and the male of the smaller deer species (hog deer and sika deer), and with males generally being the larger sex this hybridization pattern may be reflective of the phenotypic limitations that the reciprocal cross would present. However, this may be less of a consideration between hog deer and chital as overall sizes are relatively similar, and crosses have been reported to occur both ways (Gray, 1972). Hybrids between hog deer and chital also appear to favor hog deer‐like phenotypes. McMaster (1871) describes hybrids as having similar behavioral characteristics as hog deer, and with darker fur and fainter spots than chital, while Gray (1972) reported a hybrid that resembled hog deer in “head, face, and horns,” with a white‐spotted coat resembling chital. It is also possible that backcrossing to a parental species has occurred following the hybridization event, which may further explain the hog deer appearance seen in the current population. Furthermore, the presence of a shared nuclear haplotype between Victorian hog deer and *Axis porcinus* at the αLalb gene provides further support for hybridization between the two species of deer.

This study is the first to report on long‐term persistence of chital and hog deer hybrids in a wild setting. It is important to note, however, that it is unknown when the initial hybridization occurred; hybridization may have arisen in the wild in either the native range or after release of both chital and hog deer in Wilsons Prom in the 1860s or may have occurred in captivity prior to release. The scenario presented here is also somewhat unusual; traditionally, hybridization occurs when the distribution of two genetically distinct populations shares overlapping ranges, mates, and produces viable offspring, with the offspring forming a hybrid zone where overlap occurs (Shurtliff, 2013). However, no pure stock of chital is present within Victoria as the population became locally extinct in the 1920s, and no pure hog deer were identified during the course of this study. This would suggest that either both parental species have been essentially bred out of existence over generations, or only hybrids were ever released into the wild. As the original parental species are no longer present, further assessment of hybridization within Victoria, particularly detecting backcrossing to either species, is difficult as samples of parental species that contributed to the hybridization are needed for more in‐depth analysis.

The continued survival of the hybrid population in Victoria over many generations without the presence of either parental species demonstrates that hybrids between chital and hog deer are fertile. The chromosome numbers differ in these two species (chital 2*n* = 66, hog deer 2*n* = 68; Khongcharoensuk et al., 2017; Pinthong et al., 2017); however, this is not unique to hog deer and chital, with other species known to hybridize in the family Cervidae also comprising different chromosome numbers (Bonnet‐Garnier, Claro, Thevenon, Gautier, & Hayes, 2003; Gustavsson & Sundt, 1968). Robertsonian translocations of chromosomes, whereby the fusion of whole arms of two acrocentric chromosomes occurs, are common in cervids (Bonnet‐Garnier et al., 2003; Huang, Chi, Nie, Wang, & Yang, 2006), and it is likely that the prevalence of these chromosome translocations has assisted in the production of fertile hybrids where chromosome numbers are different. Robertsonian translocations have been detected in red deer and sika deer hybrids (Herzog & Harrington, 1991), and it is feasible that further investigation of hybrid hog deer and chital through karyotyping will reveal similar mutations that have made fertile hybrids possible.

The mitochondrial D‐loop region was discovered to be monomorphic within the Victorian hog deer population, suggesting that the diversity at this region of the mitochondrial genome is very low. Similar findings have been reported for other species introduced to Australia belonging to the Order Artiodactyla; mitochondrial analysis of Banteng revealed that this species was monomorphic at one mitochondrial gene and two nuclear genes (Bradshaw et al., 2006), and analysis of the D‐loop of Australian populations of dromedary camels (*Camelus dromedarius*) revealed only 13 haplotypes, which was considered low by the authors as the founder size of dromedary camels was <5,000 individuals, and their population size is now considered to be greater than 1 million animals (Spencer et al., 2012). Many more D‐loop haplotypes were observed in the native chital, *A. porcinus* and *A. p. annamiticus* populations, which is unsurprising as the Victorian hog deer population has undergone a bottleneck following introduction to Australia, and the lack of variation may leave the population vulnerable to stochastic events. However, as genetic diversity was only measured using a single mitochondrial gene, the addition of more samples and faster evolving nuclear markers will likely elucidate a better understanding of the genetic diversity within this population. The D‐loop results also show that the two samples of *A. p. annamiticus* sequenced for this study appear to be genetically distinct from all other reported *A. p. annamiticus* samples present in the haplotype network. These samples are the first *A. p. annamiticus* individuals to be sequenced from Cambodia, and all other samples present in GenBank are reported to have been sourced from Thailand, where populations became extinct in the 1960s but have since been reintroduced from unknown stock (Brook et al., 2015; Humphrey & Bain, 1990; Maxwell et al., 2006). These distinct *annamiticus* haplotype differences between Cambodia and Thailand warrant further research to ascertain whether the Cambodian *A. p. annamiticus* haplotype is distributed elsewhere and is in need of conservation intervention.

Despite low reported genetic diversity which is suggested to negatively impact populations, the initial population size of hog deer has expanded considerably since their release in Victoria. This may be explained by the enemy release hypothesis, whereby an introduced species becomes abundant and a successful invader as their population sizes are no longer affected by their native predators or pathogens (Keane & Crawley, 2002). In their native range, hog deer are an important prey item for many species, including leopard (*Panthera pardus fusca*), clouded leopard (*Neofelis nebulosa*), Burmese python (*Python bivittatus*), Bengal tiger (*Panthera tigris tigris*), and dhole (*Cuon alpinus*), and so, liberation from these predators is likely to positively impact abundance of the hog deer in Victoria (Dhungel & O'Gara, 1991; Grassman, Tewes, Silvy, & Kreetiyutanont, 2005; Prasanai, Sukmasuang, Bhumpakphan, Wajjwalku, & Nittaya, 2012; Wegge, Odden, Pokharel, & Storaas, 2009). Alternatively, there may have been some genetic fitness associated with the population, particularly immediately after hybridization. Heterosis, or “hybrid vigor,” may have made initial establishment of the hog deer in Victoria much easier than if pure stock alone had been introduced. Hybridization introduces many novel alleles into the population with which natural selection can act upon, thereby increasing overall fitness (Weeks et al., 2011; Whiteley, Fitzpatrick, Funk, & Tallmon, 2015). Hybrid vigor has been implicated in the successful introductions of many plant species (Durand et al., 2002; Moody & Les, 2002) and is now being recognized as advantageous in several animal species as well (Drake, 2006; Facon, Jarne, Pointier, & David, 2005). However, as a small founding population was established and no additional hog deer were introduced to the main population following initial release, these potential genetic benefits are likely no longer affecting the persistence of the Victorian population.

Although genetic variation is reported to be low at the mitochondrial regions in the Victorian hog deer population, it may still be worthwhile to use this population as a source for genetic rescue. Due to the discovery of hybridization with chital, translocation should be restricted to areas where both species are present in the northern regions of India. It is currently unknown how prolific hybridization is between hog deer and chital in their native range, but considering that the two species share overlapping ranges, the existence of a hybrid zone is probable. Alternatively, if natural hybrid zones are not detected in the native range after extensive study, this may give further credence to the idea that hybrids between the two species only occur in captivity and, as such, would narrow down the possibilities of where hybridization most likely occurred in the Victorian population. Often, the most significant concern cited when attempting genetic rescue is the possibility of introducing outbreeding depression in the population, whereby the offspring of parents with distinct genetic differences show a decrease in overall fitness, as they are no longer well adapted to their current environment. Currently, there is a shift away from the belief that translocation of distinct populations will lead to outbreeding depression and “genetic swamping” of locally adapted alleles (Frankham, 2015; Weeks et al., 2011). The introduction of eight Texas panthers (*Puma concolor stanleyana*) to the declining population of Florida panthers (*Puma concolor coryi*) was shown to positively affect the survival rate in hybrid panther offspring, while birth rates and pup survival increased after crossing distinct lineages of Mexican wolves (*Canis lupus baileyi*), demonstrating that some fitness advantages can be observed when crossing distinct populations (Fredrickson, Siminski, Woolf, & Hedrick, 2007; Pimm, Dollar, & Bass, 2006). The long‐term persistence of the hybrid population in Victoria suggests that outbreeding depression was not a significant factor that hindered population expansion when these crosses first occurred.

Recent genetic analysis has revealed that although hog deer have declined in their native range, this is not necessarily reflected in their genetic diversity, particularly in India (Gupta et al., 2018). Gupta et al. (2018) report moderately high levels of genetic diversity within the major Indian populations at both mitochondrial and microsatellite markers; however, lower levels are reported within Manipur, a trend which the authors attribute to fragmentation of this population. It is important to note that in this study, Gupta et al. (2018) have conducted a considerable amount of their sampling within the most abundant remnant populations of *A. porcinus*, which is reflective in their results and not necessarily representative of all extant populations of hog deer in India. Multiple mitochondrial haplotypes of hog deer are also reported in Pakistan, of which very little information is known about their current abundance and distribution (Abbas et al., 2017; Timmins et al., 2015). While the occurrence of greater diversity is evident in extant populations with higher levels of abundance, a number of native populations of hog deer are numbered in the hundreds, and local extinctions have been reported in up to 35 localities in India alone, which indicates that a number of populations may still be in need of intervention (Timmins et al., 2015). Additionally, it may be more beneficial to use individuals that have been sourced from Victoria rather than the native range, so as not to reduce abundance in the few relatively large remaining populations in India. Ultimately, while high diversity is currently present in the most abundant remaining populations in the native range, the Victorian population still likely represents an important safeguard to any future native declines.

Ultimately, translocations of hog deer require knowledge of both the source population and the receiver population. High priority should now be given to further sampling of hog deer (both *A. porcinus* and *A. p. annamiticus*) throughout their native range owing to their continued decrease in abundance; sites from India and Thailand have been sequenced in the past, but hog deer are known to occur in a number of countries including Pakistan, Nepal, Bangladesh, Bhutan, and Cambodia with little to no genetic assessment of these populations (Timmins et al., 2015). Future research within the native range should focus on the connectivity of hog deer across the native landscape and identifying the subspecies boundary between *A. porcinus* and *A. p. annamiticus*, with an overarching goal of promoting genetic diversity and effective management of hog deer across South‐East Asia. Hog deer have also previously been reported in China, Myanmar, Viet Nam, and Laos; however, it may be locally extinct in these areas, so monitoring in habitats where hog deer have previously occurred in these countries is needed to firmly establish whether local extirpation has occurred. Additionally, an investigation into the possibility of a natural hybrid zone between hog deer and chital in India is necessary, as this will likely dictate whether translocation of Victorian hog deer is suitable for genetic rescue of the species in India. Further analysis of the Victorian population of hog deer is also warranted; this study was unable to examine the genetic diversity of Victorian hog deer in‐depth as the D‐loop region of the mitochondria within this population was monomorphic. Moreover, the nuclear markers chosen for this study were shown to provide low discrimination power when comparing hog deer and chital sequences. The inclusion of polymorphic nuclear STR or SNP analysis would likely address these lingering questions and could also be used for monitoring hog deer populations pre‐ and post‐translocation, to understand the long‐term effects of using Victorian hog deer hybrids as a source for genetic rescue within the hog deer native range.

## CONFLICT OF INTERESTS

The authors declare that they have no conflict of interest.

## AUTHOR CONTRIBUTIONS

EH, AL, ST, NM, and JS contributed to the design of the study; ST and EH arranged collection of Australian samples; EH arranged collection of Cambodian samples; EH performed the laboratory procedures; EH conducted the analyses; EH led the writing of the manuscript with input from NM and JS; AL, ST, NM, and JS provided feedback and revisions of the manuscript. All authors contributed equally to the drafts and gave final approval for publication.

## Data Availability

Data are available in GenBank under accession no. MN226858–MN226880.
